# Recent advances in the application of [2 + 2] cycloaddition in the chemical synthesis of cyclobutane-containing natural products

**DOI:** 10.1007/s13659-024-00457-9

**Published:** 2024-06-11

**Authors:** Song-Yu Hou, Bing-Chao Yan, Han-Dong Sun, Pema-Tenzin Puno

**Affiliations:** Key Laboratory of Phytochemistry and Natural Medicines, Kunming Institute of Botany, University of Chinese Academy of Sciences, Chinese Academy of Sciences, Kunming, 650201 Yunnan People’s Republic of China

**Keywords:** [2 + 2] Cycloaddition, Total synthesis, Natural products, Cyclobutane

## Abstract

Cyclobutanes are distributed widely in a large class of natural products featuring diverse pharmaceutical activities and intricate structural frameworks. The [2 + 2] cycloaddition is unequivocally the primary and most commonly used method for synthesizing cyclobutanes. In this review, we have summarized the application of the [2 + 2] cycloaddition with different reaction mechanisms in the chemical synthesis of selected cyclobutane-containing natural products over the past decade.

**Graphical Abstract:**

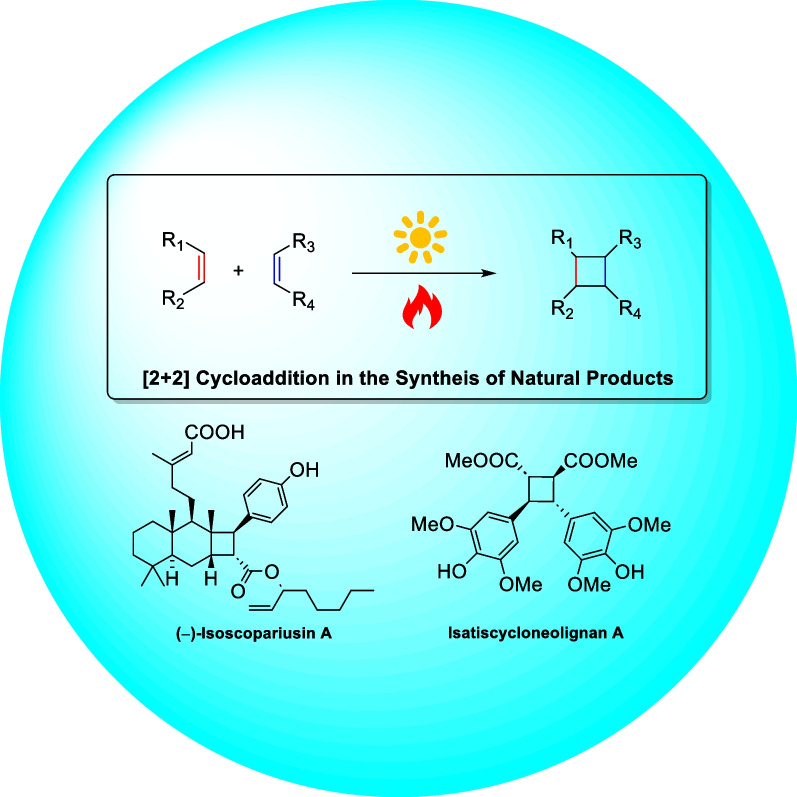

## Introduction

**Scheme 1:**
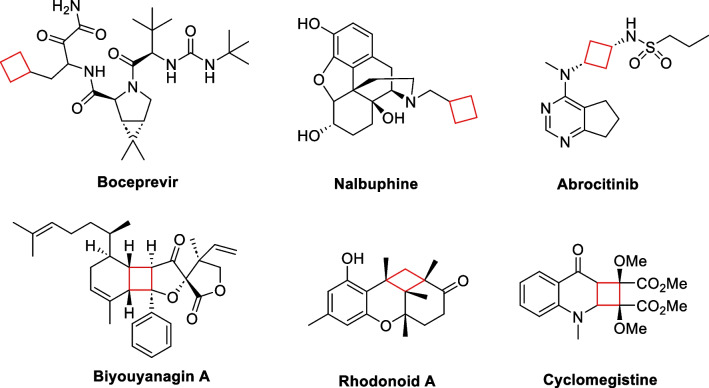
Representative drugs and natural products containing cyclobutane motifs

Natural products are an important source for the discovery of candidates of bioactive drug molecules and lead compounds. The cyclobutane subunit is prevalent in various drugs and drug prototypes such as boceprevir [1, 2], nalbuphine, exhibiting distinctive structures and broad bioactivities such as antibacterial, anti-viral, and immunosuppressant properties. In recent years, a variety of natural products containing cyclobutane motifs have been identified as terpenoids, meroterpenoids, and alkaloids. Notably, the cyclobutane-containing secondary metabolites are biosynthesized by a range of organisms from land plants to marine life forms, which indicates the importance of cyclobutane in biological evolution. Furthermore, these structures exhibit diverse biological activities with potential medicinal value (Scheme 1).

Cyclobutane motifs serve as crucial synthetic building blocks and functional groups in the modulation and design of structures in medicinal chemistry. To enhance clinical efficacy and improve ADMET properties, many pharmaceuticals have incorporated cyclobutane motifs. In 2012, Pfizer successfully launched Xeljanz, a highly potent medication targeting the JAK1/JAK3 receptors for the treatment of rheumatoid arthritis. Abrocitinib, a sulphonamide derivative approved by FDA for marketing in 2023, was developed by replacing the piperidine structure in Xeljanz with a 1,3-disubstituted cyclobutane structure, which was found to increase the selectivity by 28 times towards the JAK1 receptor in the treatment of JAK1-mediated autoimmune diseases.

Several methodologies are employed for synthesizing cyclobutane-containing natural products, such as direct intramolecular ring closure [3–8], free radical cyclization [4, 5, 9–15], [2 + 2] cycloaddition reactions [16–21], ring-expansion approaches [22, 23], ring-contraction strategies [24–26], and rearrangement reactions. These methods are proven to be effective and reliable in producing high-quality results. Among the strategies, [2 + 2] cycloaddition reactions, widely employed in organic synthesis, have emerged as the approach for cyclobutane synthesis. In recent years, significant advancements in reaction conditions have led to milder reaction conditions and improved compatibility with a broader range of substrates. This progress has expanded the scope of cyclobutane derivatives, facilitating access to a diverse array of molecules. In this paper, we make a classification of [2 + 2] cycloaddition reactions by mechanism and showcase recent successful applications of this strategy in the total synthesis of cyclobutane-containing natural products and divide them into four categories.

## [2 + 2] Cycloaddition reactions under thermal conditions

Pericyclic reactions, which are governed by the principles of molecular orbital symmetry, maximum overlap, and energy similarity, are well-understood thanks to the Woodard-Hoffmann rule and front-line orbital theory (FMO) [27]. Energetically, the two olefinic substrates are in the lowest state, where the molecular orbitals are symmetrically distributed around the carbon–carbon double bond. When coming in close proximity, cis-addition is a forbidden pathway in symmetry, resulting in an uneven reaction [28]. In contrast, the trans-addition approach can meet the requirements in symmetry, but the distorted stereochemistry still makes this issue a dilemma. In recent years, the emergence of substrates with orthogonal π-orbitals has reversed this trend [20, 29–31], allowing for reactions to occur under standard conditions with confidence. Currently, substituted cyclobutane motifs can be synthesized through two main methods in this condition. One is the addition of olefinic substrates to ketene [19, 32–40] or keteniminium ions [41–49] to produce cyclobutanone, followed by multi-stage functional group transformations. This approach has been proven to be highly effective and reliable. The other is the addition of olefinic substrates to allenes, leading to the efficient formation of cyclobutane adducts [50–56].

In 2021, Puno’s group successfully completed the total synthesis of ( −)-isoscopariusin A [57], a meroditerpenoid isolated from the aerial parts of *Isodon scoparius*. This compound features a unique 6/6/4 tricyclic skeleton and a tetrasubstituted cyclobutane motif. Due to the crowded cyclobutane in geometry and numerous stereochemical centers, the synthesis of this molecule is challenging. Bioactivity studies suggest that this molecule has significant immune-suppressive activity in T-cell proliferation. The synthetic route commenced with sclareolide as the starting material, leading to the production of compound **1** through six steps of reactions. Subsequently, thermal [2 + 2] cycloaddtion between **1** and dichloroketene, in situ generated by zinc dust and trichloroacetyl chloride, resulted in the formation of dichlorocyclobutanone **2**. Treatment of **2** with samarium diiodide removes the chlorine atom, yielding the cyclobutanone **3**, followed by the Shapiro reaction to give the unsaturated ester **4**. Compound **4** reacted with Grignard reagent to introduce an aryl side-chain into the cyclobutane motif, yielding compound **5** and completing the overall synthesis of the molecule in 24 steps involving configuration inversion, Ni-catalyzed cross-coupling and deprotection.

**Scheme 2:**
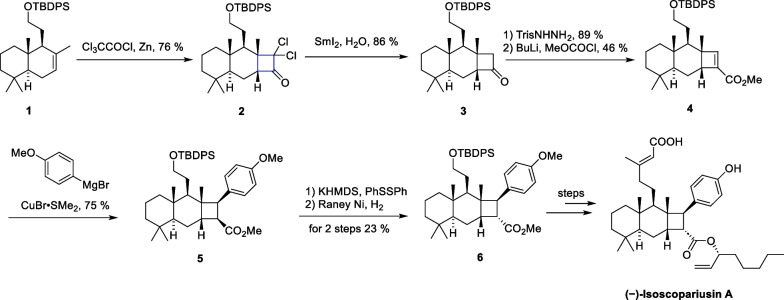
[2 + 2] cycloaddition between alkene and ketene in the synthesis of ( −)-isoscopariusin A

**Scheme 3:**
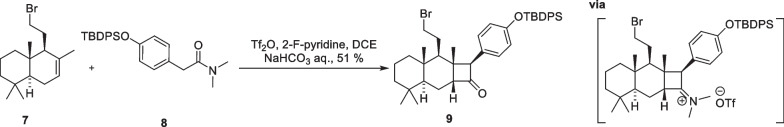
[2 + 2] cycloaddition between alkene and keteniminium ions in the synthesis of ( −)-isoscopariusin A

**Scheme 4:**
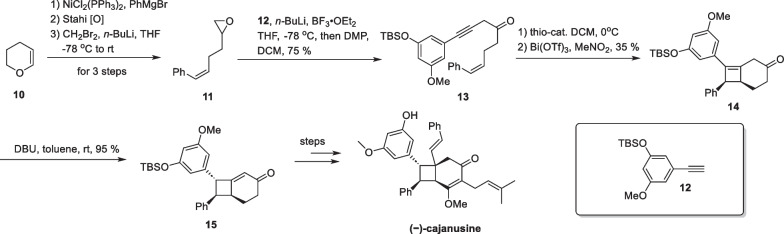
Intramolecular [2 + 2] cycloaddition between alkene and allene in the synthesis of ( −)-cajanusine

In the first route for the synthesis of 6/6/4 tricyclic core, configuration inversion of cyclobutane with mismatched C-8′ significantly lowered the overall efficiency. The authors have successfully developed a strategy to synthesize an *α*-arylcyclobutanone followed by face-selective homologation to access the key tricyclic core (Scheme 2). Inspired by the pioneering work of Ghosez et al. on the development of keteniminium ions chemistry [46–48, 58, 59], compound **8** was obtained in a single step by an intermolecular [2 + 2] thermal cycloaddition between alkene **7** and keteniminium salts. This intermediate was subsequently transformed through carbon chain extension, Ni-catalyzed cross-coupling, and other functional group transformations, ultimately leading to a 12-step synthesis of ( −)-isoscopariusin A on a gram scale. The [2 + 2] cycloaddition of olefins with ketene or keteniminium salts shows notable reactiveness, robustness, and applicability, demonstrating the potential for broad application (Scheme 3).

( −)-Cajanusine, a styrene-type compound with a fully substituted cyclobutane, was isolated from the leaves of *Cajanus cajan* by Ye’s group [59]. The molecule exhibits significant cytotoxic activity against HepG2 and HepG2/ADM cells. Brown’s group completed its first asymmetric total synthesis in 2020 [60] (Scheme 4).

**Scheme 5:**
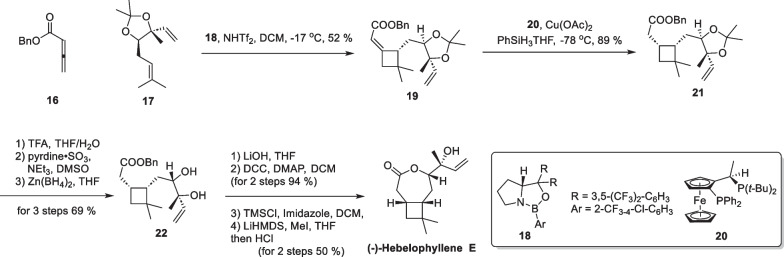
Total synthesis of ( −)-hebelophyllene E

**Scheme 6:**
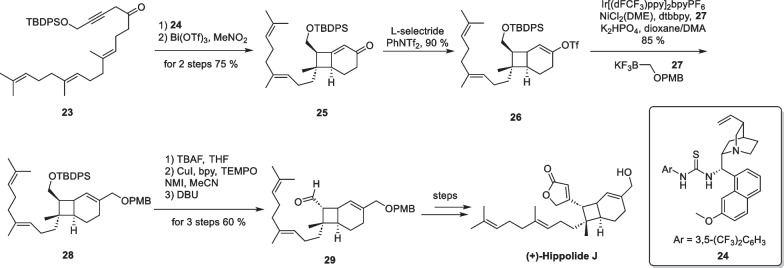
Total synthesis of ( +)-hippolide J

They employed an enantioselective [2 + 2]-cycloaddition strategy to construct a cyclobutane motif from allene and olefin. Starting with dihydropyran **10**, the authors conducted a coupling reaction with phenyl Grignard reagent under Ni-catalyzed conditions [61]. In the presence of butyl lithium, the acetylide was obtained from compound **12** and then underwent addition to epoxide **11**, followed by oxidation to produce compound **13** in 75% yield. Employing a chiral thiourea catalyst enables compound **13** to undergo an enantioselective isomerization reaction, generating an allenic ketone in situ, which undergoes a [2 + 2] cycloaddition reaction with an intramolecular double bond to give compound **14**, with Bi(OTf)_3_ activating the carbonyl group near to the allene [62].

( −)**-**Hebelophyllene E, a novel sesquiterpene containing cyclobutane motif, was isolated from the ectomycorrhizal fungus *Hebeloma longicaudum* in 1999 [63]. Notably, determining the absolute configuration of the tertiary alcohol structure on the side chain using conventional spectroscopic methods proved to be challenging. In 2018, Brown successfully carried out the total synthesis and structural elucidation [52] (Scheme 5). The synthesis commenced with two prepared reaction substrates, compounds **16** and **17**, on a decagram scale. Through extensive screening of various reaction conditions, a cyclobutane adduct **19** was assembled by an intermolecular asymmetric [2 + 2] cycloaddition reaction using a chiral CBS catalyst. Subsequent reduction of the unsaturated ester in a one-step MHAT reaction [64] afforded **21** with preserved stereochemistry in the use of ligand **20**. Trifluoroacetic acid was employed to remove the protection group of **21**, followed by stereochemical reversal of the hydroxyl group through a tandem redox process and lactonization reaction, enabling a successful total synthesis of ( −)**-**hebelophyllene E in 10 steps.

( +)-Hippolide J, a marine natural product with potent antifungal activity, was discovered in 2017 by Lin’s group from the marine sponge *Hippospongia lachne* [65]. The fully substituted cyclobutane motif presents significant challenges from a synthetic standpoint. In 2020, Brown’s group accomplished the first asymmetric total synthesis of this molecule utilizing an intramolecular [2 + 2] cycloaddition reaction between allene and alkene [66] (Scheme 6). Compound **23** was prepared in five steps, starting from farnesyl acetone before being subjected to isomerization in the presence of thiourea catalyst **24**, resulting in an enantioselective yield of an allene intermediate. The intermediate then underwent an intramolecular [2 + 2] cycloaddition reaction, under the catalysis of Bi(OTf)_3_, ultimately producing compound **25**. Subsequent reduction of **25** followed by being captured by the Comins reagent to produce **26**. The authors utilized the reaction conditions of photoredox and nickel dual catalysis developed by Molander and co-workers [67, 68]. Compound **28** was synthesized through a one-step coupling reaction between compounds **26** and **27**. Finally, the adduct was deprotected and subjected to oxidative tandem lactonization to complete the total synthesis of ( +)-hippolide J.

## [2 + 2] Cycloaddition reactions under photocatalytic conditions

### Direct photosynthesis

E.J. Corey discovered the generation of cyclobutane fragments during the synthesis of the sesquiterpene compound caryophyllene [69]. In the presence of excess isobutene, the *α,β*-unsaturated ketone experienced a direct intermolecular [2 + 2] reaction to produce a cyclobutane linked to cyclohexane under photocatalytic conditions. Despite the presence of regional isomers of “head-to-head” and “head-to-tail” adducts and the low yields, this study has revealed the potential applications of photochemical [2 + 2] cycloaddition reactions in total synthesis.

**Scheme 7:**
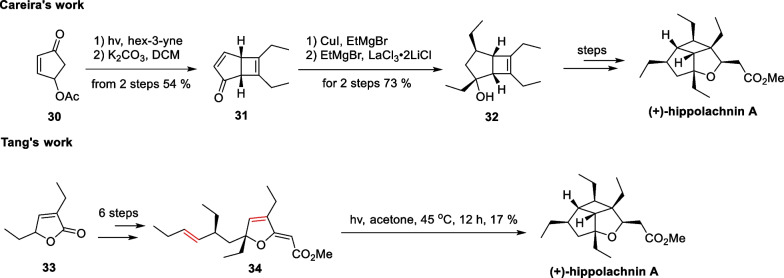
Total synthesis of hippolachnin A

Hippolachnin A, a polyketide natural product with a distinctive 5/5/4 tricyclic bowl structure, was isolated from a sponge in the South China Sea by Lin’s group in 2013 [70]. Structurally, this molecule possesses six consecutive stereocenters densely distributed on the backbone, four of which bear an ethyl substituent that protrudes in the convex direction, giving rise to a solid cage-like skeleton. Since its discovery, many synthetic chemists have pursued the total synthesis of this molecule. Since 2015, Carreira [71], Tang [72], and Trauner [73] have completed the total synthesis study of this molecule. The construction of the poly-substituted cyclobutane fragment was accomplished using the photocatalytic [2 + 2] reaction as a key step. Ding et al. have covered the synthetic efforts towards this molecule in their review [74] (Scheme 7).

**Scheme 8:**
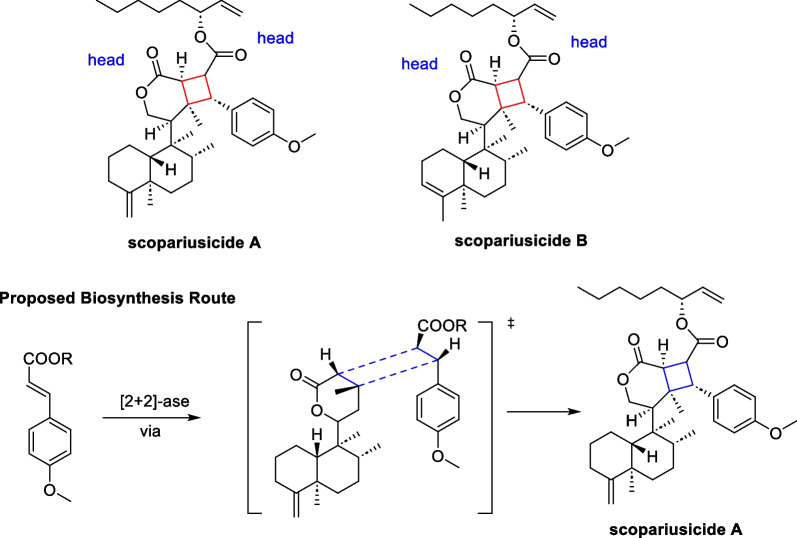
Scopariusicides A, B and its proposed biosynthesis

In the course of systematic studies of *Isodon scoparius*, Puno’s group discovered two cyclobutane-containing meroterpenoid scopariusicides A and B in 2015 [75]. The two molecules represent a new class of meroditerpenoids with unsymmetrical cyclobutane motifs and ten stereochemical centers, three of which are quaternary (Scheme 8). These structural features give the cyclobutane structure a high degree of ring tension as well as spatial site resistance, making the synthesis of this compound more challenging. In the bioactive studies, scopariusicide A also showed excellent inhibition against human T cell proliferation in vitro at a very low IC_50_ (20.7 μM), which is significantly better than the positive control BD750. The authors proposed a biogenetic pathway for scopariusicides via a crossed “head-to-head” intermolecular [2 + 2] cycloaddition between an *ent*-clerodane diterpenoid **35**, a high-content constituent in *I. scoparius*, and an unusual derivative of *trans*-4-hydroxycinnamic acid.

**Scheme 9:**
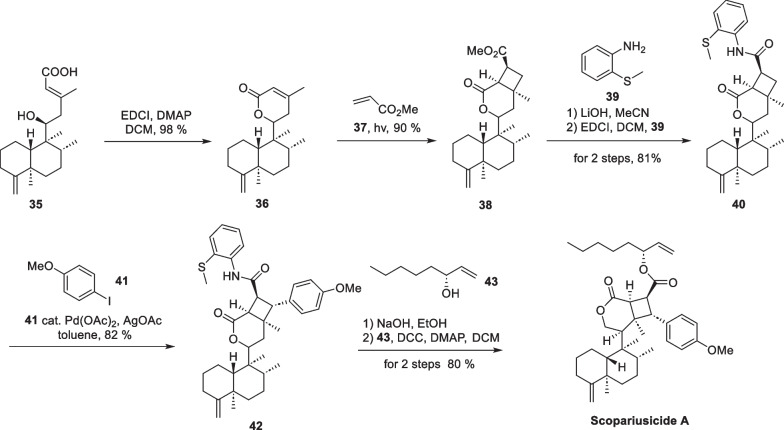
Total synthesis of scopariusicide A

In the biomimetic synthesis of scopariusicide A (Scheme 9), compound **35**, an abundant *ent*-clerodane in *I. scoparius*, was taken as the starting material, and underwent lactonization and isomerization to give **36**. Subsequently, **36** underwent an intermolecular [2 + 2] cycloaddition with methyl acrylate to yield the adduct **38** in a “head to head” form under direct UV irradiation. Compound **38** underwent immediate hydrolysis under alkaline conditions to give the corresponding carboxylic acid product, followed by amidation to give compound **40**. The amide group in **40** served as a substrate for Pd-catalyzed C-H activation to introduce an aryl ring in the cyclobutane fragment. Daugulis succeeded in arylation and alkylation of sp2 and sp3 C-H bonds using the amide group as the directing group and Pd(OAc)_2_ as the metal reagent [76, 77] in 2005 and 2010. Subsequently, Baran [78], Maimone [79], and Chen [80] et al. applied this strategy to the total synthesis of complex natural products. In this work, the introduction of aryl fragment of cyclobutane was also carried out using the strategy of C-H activation. In the synthesis of compound **42**, the amide carbonyl group of **40** directed palladium acetate to the C-H bond in the *β*-position, which was subsequently followed by transmetallation and reductive elimination with compound **41** to introduce an aryl side chain, exhibiting excellent chemoselectivity and stereoselectivity. Subsequent removal of the directing group of **42** and an esterification reaction with (*R*)-1-octene-3-ol **43** allowed for a successful total synthesis of scopariusicide A. The combination of the crossed intermolecular [2 + 2] cycloaddition and the directing Pd-catalyzed C-H activation provides an important foundation for the total synthesis of other natural products with polysubstituted cyclobutane moieties.

**Scheme 10:**
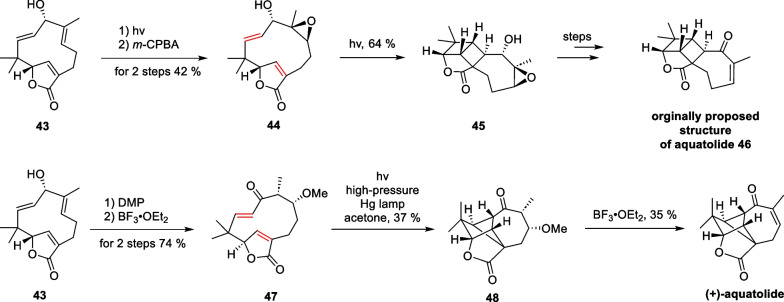
Total synthesis of aquatolide by Takao

Aquatolide is a representative sesquiterpenoid isolated from *Asteriscuc aquaticus* in 1989 by San Feliciano et al. [81] Structurally, the compound possesses a specialized bicyclo[5.1.1]nonane and bicyclo[2.1.1]hexane backbone, which presents a very significant synthesis challenge. In 2019, Takao et al. accomplished a biomimetic synthesis of quatolide with a late-stage intramolecular transannular [2 + 2] cycloaddition as a key strategy [82] (Scheme 10). This synthetic work commenced with a starting material **43**, which can be transformed into the initially proposed structure, through multiple steps including direct intramolecular [2 + 2] cycloaddition in 1989. Further oxidation of compound **43** leads to yield compound **47**. Afterward, a [2 + 2] cycloaddition reaction was carried out, followed by the removal of the protecting group, resulting in the formation of aquatolide [83].

**Scheme 11:**
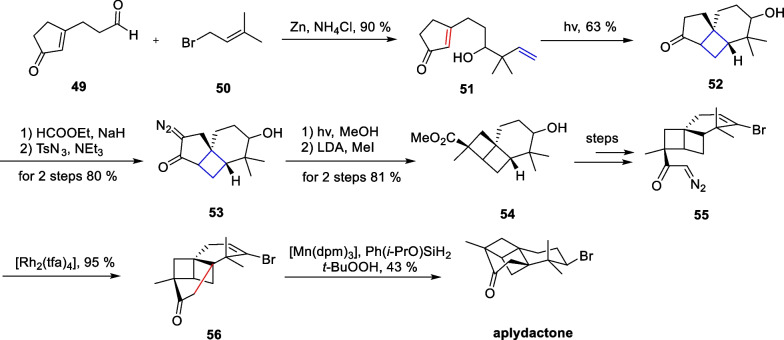
Total synthesis of aplydactone by Zhang

Marine natural products have emerged as a research hotspot in recent years, attracting the interest of chemists worldwide. In 2001, Stonik and co-workers isolated a halogenated sesquiterpene named aplydactone [84], which possessed an extremely tensile cyclobutane motif and a bromine-containing chiral center. In addition, the three quaternary carbon centers of the molecule are located at the junctions of the four-membered ring, which results in the susceptibility of the molecule to ring system rearrangements under acidic conditions. The true structure of the compound was not determined until 15 years later using single-crystal diffraction. Therefore, this molecule is extremely challenging to synthesize. In 2017, Zhang and co-workers from Xiamen University accomplished the total synthesis of this molecule, using intramolecular [2 + 2] cycloaddition and carbene insertion reactions as key strategies [85] (Scheme 11).

A selective allylation of **49** with a nucleophilic attack by **50** afforded compound **51**. Subsequently, an intramolecular [2 + 2] cycloaddition reaction can be carried out under photocatalytic conditions to yield compound **52**. A formylation/diazotransfer process produces the α-diazoketone **53**, followed by a Wolff rearrangement leading to the ring contraction to produce compound **54**. In the presence of [Rh_2_(tfa)_4_], the diazoketone structure of compound **55** is denitrated to produce carbenes intermediate, which then interact with the catalyst to form a metal carbene intermediate, undergoing a one-step carbene insertion reaction on the neighboring C-H to construct the high-tensile bridged ring. The brominated double bond in the obtained **56** was reduced by the MHAT reaction [64] to produce the natural product aplydactone.

### Photocatalytic energy transfer strategy (EnT)

As discussed earlier, direct photosynthesis has broad applications in synthesizing natural products containing cyclobutane. However, this strategy has certain limitations. First, the light source is mainly a high-pressure mercury lamp and a short-wavelength UV light source to ensure smooth excitation of the carbon–carbon double bond in one of the substrates from the ground state to the spin singlet state. The high energy required for the excitation process not only promotes the electrons on the carbon–carbon double bond to high energy levels but also induces side reactions, such as geometrical configuration distortion and functional group cleavage. Second, direct photochemical [2 + 2] addition reactions tend to occur between electron-rich neutral olefins and electron-deficient olefins with a narrow substrate range. Therefore, energy transfer strategies for [2 + 2] cycloaddition reactions serve as a valuable complement to direct photochemical reactions.

**Scheme 12:**
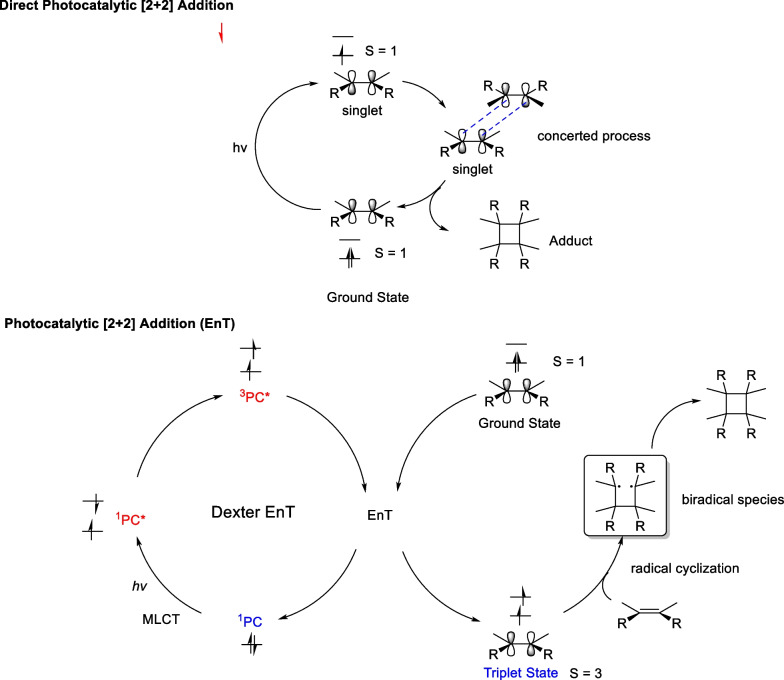
Mechanism of the direct photocatalytic [2 + 2] cycloaddition and EnT pathway

The mechanism between energy transfer strategy and direct photochemical [2 + 2] addition is quite different [86–89] (Scheme 12). In the EnT process, visible light or near-ultraviolet light is used to excite the photosensitizer to the spin-triplet state in two steps, which activates the alkenes to the triplet state in Dexter energy transfer. In recent years, transition metal complex, represented by Ir and Ru, have flourished and shown strong applications in organic chemical reactions with energy transfer strategies.

**Scheme 13:**
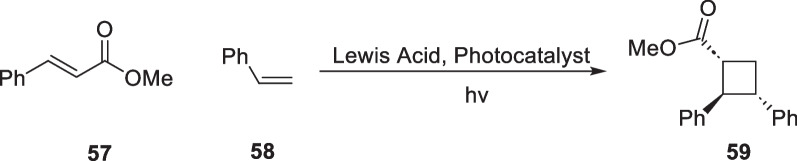
EnT mediated [2 + 2] cycloaddition by T.P.Yoon

**Scheme 14:**
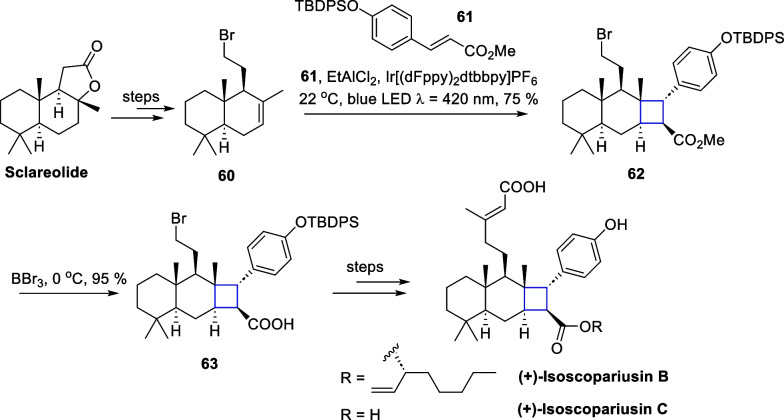
EnT mediated [2 + 2] cycloaddition in the total synthesis of ( +)-isoscopariusins B and C

In 2019, Yoon's group reported using Lewis-catalyzed energy transfer strategy to mediate intermolecular [2 + 2] cycloaddition reactions between cinnamate derivatives and styrene substrates [90], which expanded the application of this strategy to natural product synthesis (Scheme 13). In 2023, Puno and co-workers conducted a semisynthetic study of the natural products ( +)-isoscopariusins B and C [91], with EnT mediated [2 + 2] reaction as a key transformation (Scheme 14).

Starting from ( +)-sclareolide, a commercially available chiral source, **60** was obtained through hydrolysis of the lactone, reduction, bromination, and MHAT reactions developed by the Shenvi’s group [64]. Building on the groundwork of Yoon’s group in 2019 [90], screening of conditions and optimization of an intermolecular crossed [2 + 2] cycloaddition reaction were made with methyl cinnamate derivatives, affording a single diastereoisomer **62** under 420 nm blue LED illumination with dichloroethylaluminum as a Lewis acid [19]. Selective elimination of the methyl group through boron tribromide conditions yielded the carboxylic acid **63**. An esterification with **63** and a Ni-catalyzed cross-coupling reaction culminated in the synthesis of ( +)-isoscopariusins B and C. Notably, the [2 + 2] cycloaddition reaction, mediated by the EnT strategy, allows for the preparation of tetrasubstituted cyclobutane substrates on a gram scale, while preserving the halogen atoms and other functional groups. This approach offers broad substrate adaptability, compatibility with various functional groups, and mild reaction conditions.

**Scheme 15:**
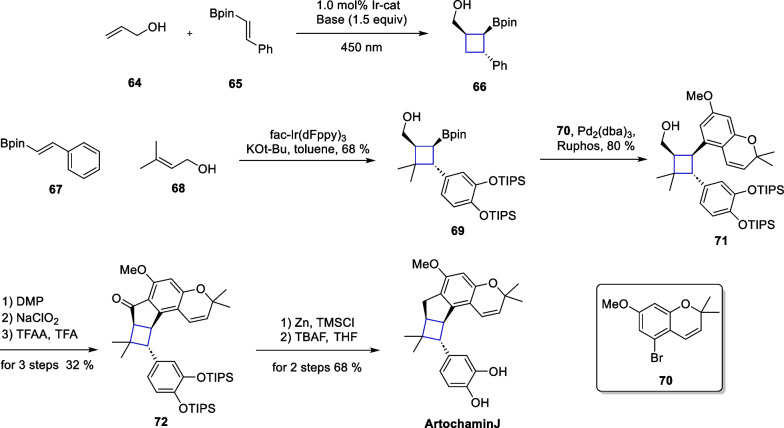
Photocatalytic [2 + 2] cycloaddition in the total synthesis of artochamin J

Among the various natural products containing cyclobutane, artochamin J and piperarborenine B are two representative molecules. In scheme, Wang et al. discovered a fascinating structure named artochamin J [92], in this class from a root extract of the plant. The molecule has bicyclo[3.2.0]heptane carbon frameworks, as well as four consecutive stereocenters on cyclobutane motif, which greatly increase the synthetic challenges. The molecule was isolated and obtained as a pair of enantiomers, suggesting that the formation of cyclobutane may occur through a non-enzymatic reaction. Piperarborenin B, an amide compound, contains a densely packed and fully substituted cyclobutane motif [93]. In 2022, Brown introduced a [2 + 2] reaction methodology in a transient coordination mode with high functional group compatibility and high reaction yields, facilitating the total synthesis of these two molecules [94] (Scheme 15).

In this study, the authors utilized compounds **64** and **65** as template substrates. They screened photocatalysts, bases, and reaction solvents to find the optimal reaction conditions. As a result, compound **66** was prepared rapidly in a yield of up to 89% and a diastereoselectivity exceeding 20:1. After successfully realizing the transformation of the template reaction, an extension of the substrate scope was pursued. The authors screened more than thirty substrates, all of which demonstrated the robustness and universality of the method. Moreover, the authors also provide insights into the reaction mechanisms. Under alkaline conditions, the hydroxyl group can be deprotonated to form an alkoxy-negative ion, and this species can subsequently coordinate with the boron atom. This transient coordination spatially closes the distance between the two substrates, and the photocatalyst then sensitizes the conjugated olefin to the triplet state, where an intermolecular [2 + 2] cycloaddition reaction takes place. The authors synthesized compound **69** using compounds **67** and **68** through an intermolecular [2 + 2] cycloaddition mediated by the energy transfer strategy (EnT). Subsequently, a Suzuki reaction of **69** with compound **70** produced compound **71**. Further oxidation and Clemmensen reduction in tandem remove the TIPS protecting group to give artochamin J.

**Scheme 16:**
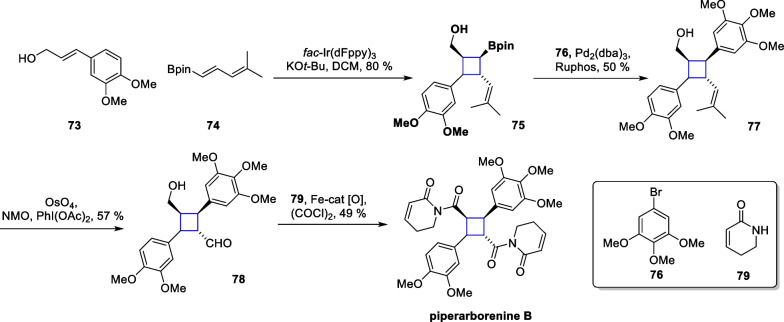
Total synthesis of piperarborenine B

The method was further utilized to synthesize piperarborenine B. Commercially available compounds **73** and **74** were subjected to light to produce adduct **75**, which subsequently underwent a Suzuki coupling reaction with **76** to produce bis-aryl-substituted product **77**. Compound **78** was formed through a cleavage reaction by OsO_4_, followed immediately by an oxidative tandem amidation process, culminating in the total synthesis (Scheme 16).

**Scheme 17:**
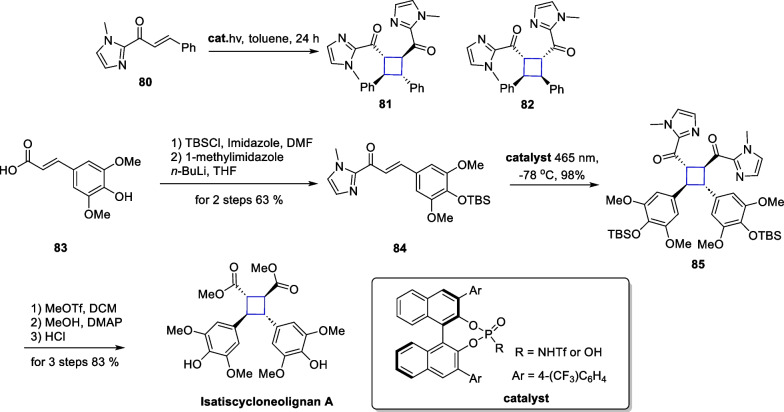
Total synthesis of isatiscycloneolignan A

Truxinates are a large class of natural products consisting of cinnamate dimers, with more than 100 reported compounds. Isatiscycloneolignan A, a highly oxidized truxinate-type compound, was isolated from the leaves of *Isatis indigotica* [95]. This compound exhibited excellent neuroprotective activity. Barbarumamide C from *Lycium barbarum* is also a representative compound of the truxinates family [96]. Biologically, this molecule shows superior anti-AD activity compared to resveratrol and is expected to be used as an adjuvant drug in the treatment of AD. In 2023, Yoon reported an intermolecular asymmetric [2 + 2] cycloaddition catalyzed by chiral Bronsted acids and completed the total synthesis of these two natural products by this approach [97] (Scheme 17).

**Scheme 18:**
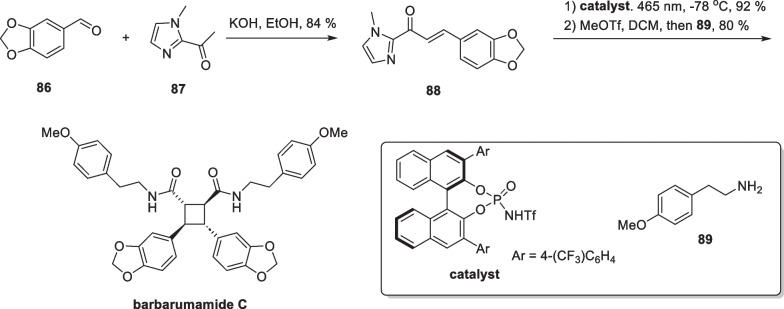
Total synthesis of barbarumamide C

The authors designed compound **80** as a substrate for the template reaction. Compound **80** yielded two addition products, **81** and **82**, with different relative configuration under light. Through optimization of the reaction conditions and screening of bronsted acids, the addition product could be obtained in 87% yield with very high selectivity (99% *e.e.*, d.r. = 11:1). Control experiments demonstrated that in the absence of a chiral bronsted acid, the addition reaction occurred with low yields and poor selectivity. The authors expanded the substrates and found that site-blocking and electronic effects had minimal impact on the reaction. The reaction was generally feasible and dominated by all-trans addition products. After demonstrating the feasibility of this strategy, the authors used it as a general method to synthesize more complex and biologically active members of this class. Compound **84** can be obtained from starting material **83** by TBS protection and subsequent amidation. Subsequently, the adduct **85** can be obtained through self-dimerization using this strategy. A subsequent 3-step transformation culminated in the total synthesis of isatiscycloneolignan A. Additionally, the authors also successfully synthesized another cinnamate derivative **88**, using an aldol reaction between compounds **86** and **87**, and then completed the total synthesis of barbarumamide C using this same strategy (Scheme 18).

**Scheme 19:**
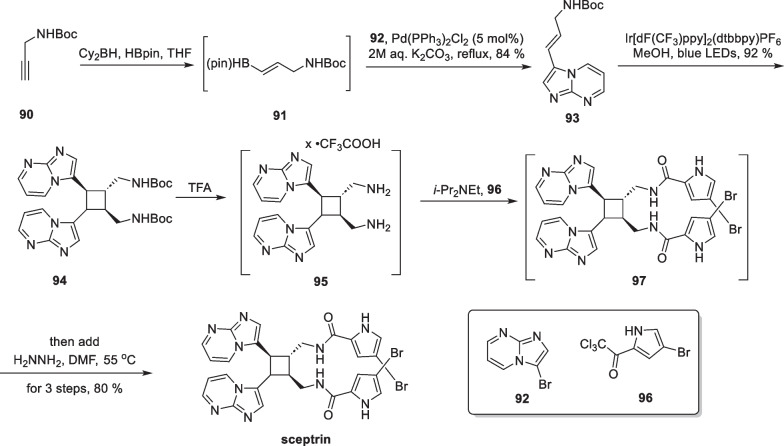
EnT mediated [2 + 2] cycloaddition in the total synthesis of sceptrin

Sceptrin, a unique pyrrole-imidazole alkaloid, was initially isolated from the Caribbean sponge *Agelas sceptrum* by Clardy, Faulkner et al. in 1981 [98], possesing a tetrasubstituted cyclobutane core. It is likely that the molecule is formed through a “head-to-head” intermolecular [2 + 2] reaction of the monomer hymenidin [99]. After its discovery, scientists conducted semi-synthetic and biomimetic synthetic studies on sceptrin. In 2020, Jamison reported the shortest synthetic route to date with a 4-step synthesis of the compound [100] (Scheme 19).

The commercially available N-Boc-propargylamine **90** was used to synthesize compound **91** through a B-H insertion reaction. They then performed a one-pot Suzuki reaction of compound **91** with compound **92** using Pd(PPh_3_)_2_Cl_2_ as a catalyst to produce **93** without purification in two tandem reactions. Compound **93** is made up of disubstituted olefins and heterocyclic fragment that can form conjugations with low excitation energies. Various photocatalysts and solvents were initially tested, leading to the discovery that Ir[dF(CF_3_)ppy]_2_(dtbbpy)PF_6_ can stimulate compound **93** to the triplet state, facilitating an intermolecular [2 + 2] cycloaddition to afford **94**. With compound **94** in hand, the Boc groups are removed using trifluoroacetic acid, resulting in the primary amine structure, which is then subjected to an amidation reaction with compound **96** to produce amide **97**. Compound **97** was synthesized by a one-step hydrazinolysis reaction, culminating in the synthesis of sceptrin. It is noteworthy that the synthetic route only involved three times of purification, and many chemical transformations were conducted using a one-pot method. The [2 + 2] cycloaddition, mediated by the energy transfer strategy, showed excellent reaction efficiency and broad functional group compatibility.

### Electron transfer strategy (SET)

**Scheme 20:**
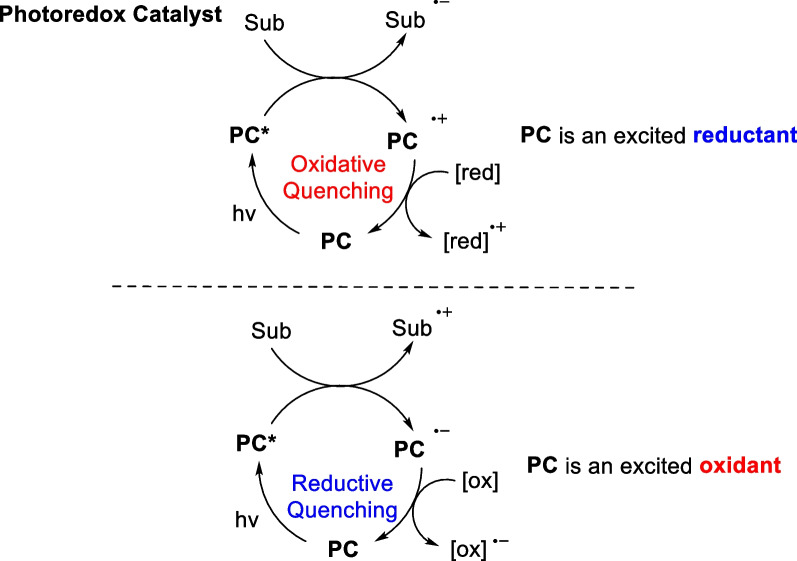
Mechanism of the [2 + 2] cycloaddition mediated by SET

Both the direct photocatalytic [2 + 2] cycloaddition reaction and the [2 + 2] cycloaddition reaction mediated by energy transfer strategy involve interactions between an excited state substrate and a ground state substrate. Although the former is a synergistic process in terms of the reaction mechanism, the latter is a step-wise process of tandem cycloaddition of free radicals, both strategies involve electroneutral intermediates that mainly occur between neutral olefins and electron-deficient olefins. For the [2 + 2] cycloaddition reactions of electron-rich olefinic substrates and electron-deficient substrates, the visible light-catalyzed electron transfer strategy provides a powerful complement [101–103]. The electron transfer approach involves using the high redox potential of the excited state transition metal complex to oxidize a single electron in the electron-rich olefinic substrate. This electron-deficient radical cation is susceptible to nucleophilic attack by another substrate double bond, leading to the formation of carbon–carbon bonds through a reductive quenching process. The electron transfer strategy can also utilize the excited state transition metal complexes as electron donors to transfer their own single electrons to the LUMO orbitals of the electron-deficient olefin to produce an active radical anion. This electron-rich species can readily undergo nucleophilic attack on another electron-deficient olefinic substrate and subsequently loses a single electron, completing the catalytic cycle to form a carbon–carbon bond [102–107] (Scheme 20).

**Scheme 21:**
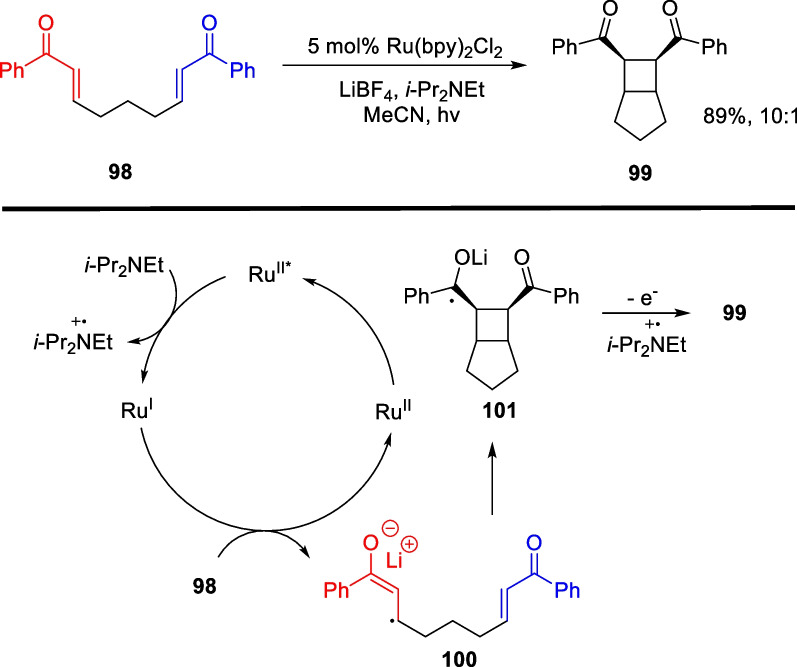
SET mediated intramolecular [2 + 2] cycloaddition by T.P. Yoon

In 2008, Yoon reported visible light-catalyzed intramolecular [2 + 2] cycloaddition reactions of double electron-deficient alkenes [108] (Scheme 21). The authors achieved a remarkable yield up to 89% and 10:1 diastereoselectivity in generating tetrasubstituted cyclobutane fragments. Using Ru(bpy)_3_^2+^ as a photocatalyst and DIPEA as an electron donor, the valence of the excited state photocatalyst was lowered, which initiated a one-electron reduction process of the photocatalyst to the electron-deficient substrate. This resulted in the production of a radical anion that underwent an intramolecular cyclization process. Yoon’s group successfully filled a gap in electron-rich olefin substrate reactions by the discovery of a [2 + 2] cycloaddition reaction between intermolecular dual electron-rich alkenes four years later [109].

**Scheme 22:**
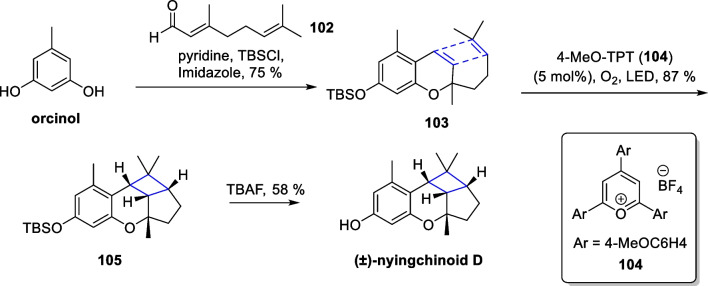
SET mediated intramolecular [2 + 2] cycloaddition in the total synthesis of ( ±)-nyingchinoid D by George

In 2018, Hou reported eight new polycyclic meroterpenoids molecules as enantiomers from *Azalea ninghaiensis*. Among them, ( ±)-nyingchinoid D [110] featured a ring system with a fused cyclobutane scaffold. Later, George’s group successfully synthesized nyingchinoid D and its homologs [111] (Scheme 22). Starting from orcinol, the authors obtained a bis-olefinic substrate **103** with tandem TBS protection by Friedel–Crafts alkylation reaction. With 4-MeO-TPT **104** as the photocatalyst and oxygen as the oxidative quencher of the photocatalyst, compound **105** was obtained in 87% yield under light. Subsequent removal of the TBS group delivered ( ±)-nyingchinoid D.

In 2007, Ronald J. Quinn and his team discovered a new lignan, endiandrin A, from the roots of *Endiandra anthropophagorum* using a bioactivity-directed isolation method [112]. Endiandrin A is supposed to comprise two monomeric molecules formed by a "head-to-head" fashion, forming a cyclobutane with all-trans substituents. This unique backbone structure is very rare in lignans. The authors conducted activity tests and structural derivatization of the molecule at the beginning of the isolation. Both this compound and its methylated derivatives, di-*O*-methylendiandrin A, showed specific antagonism against the glucocorticoid receptor and were considered a promising new class of adjuvant drugs for treating rheumatoid arthritis. Therefore, the total synthesis of this molecule is of paramount practical significance.

**Scheme 23:**
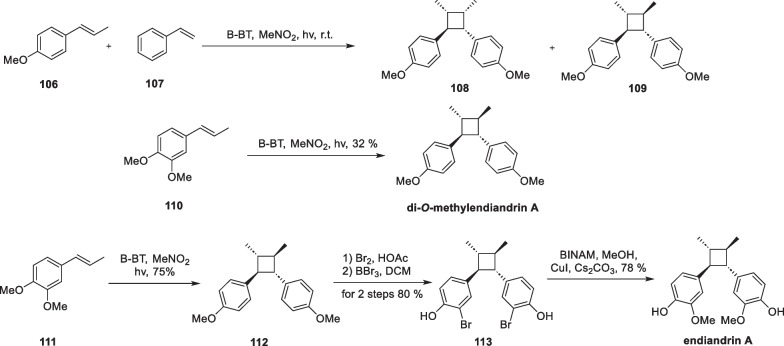
Total Synthesis of endiandrin A and di-*O*-methylendiandrin A

In 2017, Zhang developed a heterogenetic photocatalyst-mediated intermolecular crossed [2 + 2] cycloaddition reaction [113]. This strategy was employed to achieve the enantioselective total synthesis of endiandrin A and di-*O-*methylendiandrin A. Conventional organic photocatalysts, such as aromatic ketones and organometallic complexes, often coexist with the reaction substrate in the solution during the reaction. Although they can provide better energy transfer efficiencies by achieving fuller and closer contact, the substrates and products are hard to separate from these catalysts. Therefore, additional purification means are often required. To tackle this issue, the authors used a photocatalyst based on a conjugated microporous polymer network, expanding the scope and type of photocatalysis (Scheme 23).

The synthesized polymer-attached photocatalysts B-BT were characterized using scanning electron microscopy (SEM), transmission electron microscopy (TEM), and solid-state NMR techniques. The results showed that the catalysts were uniformly arranged in the form of fibers. Subsequently, the authors established a template reaction for the cycloaddition reaction using **106** and **107** as substrates and B-BT as the photocatalyst. Through screening of reaction conditions, compounds **108** and **109** were obtained in a good yield under optimal conditions. After establishing the robust reaction conditions, the authors achieved the one-step total synthesis of di-*O*-methylendiandrin A from compound **110**. Compound **111** underwent dimerization under light conditions to yield compound **112**, which was then subjected to bromination and copper-catalyzed cross-coupling, affording the target endiandrin A.

## Transition metal-catalyzed [2 + 2] cycloaddition strategy

As previously mentioned, the [2 + 2] cycloaddition reaction involves the formation of carbon–carbon bonds between a ground state olefinic substrate and an excited state olefinic substrate or radical ions. This process occurs in a synergistic or stepwise manner, as the two substrates are brought into close proximity and their molecular orbitals overlap. In addition, the substrates used for cyclization reactions require the use of olefinic substrates with extended conjugation, whether by direct excitation, triplet state sensitized activation mode or based on radical ions generated by photoredox strategies. Unconjugated aliphatic olefins generally exhibit transitions with short wavelengths (< 200 nm), higher-energy triplet excited states, and redox potentials that are beyond the scope of most common photocatalysts. For the [2 + 2] cycloaddition reaction of bis-aliphatic olefin substrates, transition metal catalysis plays a particularly important role.

In 1973, Kochi and Salomon reported that Cu(I) salts, particularly CuOTf, which has a better catalysis effect on the [2 + 2] cycloaddition process among many transition metals [114]. Under light irradiation, the Cu-olefin complex undergoes either a metal-to-olefin ligand charge transfer (MLCT) or an olefin ligand-to-Cu charge transfer (LMCT) process, which in turn induces cyclization to form a cyclobutane structure. Although the substrate scope is limited, the CuOTf-catalyzed photochemical [2 + 2] reaction successfully yielded cyclobutane-containing natural products.

**Scheme 24:**
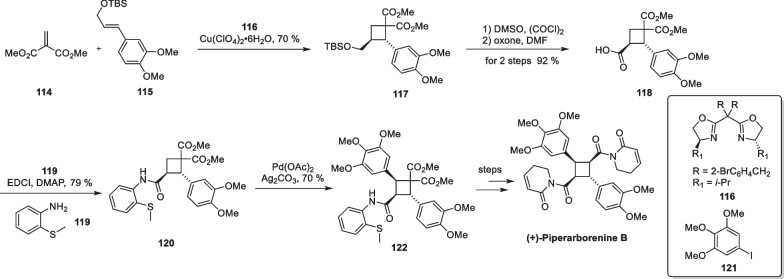
Cu(ClO_4_)_2_·6H_2_O mediated intramolecular [2 + 2] cycloaddition in the total synthesis of ( +)-piperarborenine B

In 2021, Yoon’s group accomplished the complete synthesis of advanced intermediates using this strategy [116]. Piperarborenin B is an amide compound that was isolated by Chen et al. in 2004 from the stems of *Piper arborescens* [94]. It contains a densely packed, tetrasubstituted cyclobutane fragment and exhibits excellent inhibitory effects against various cancer cell lines. This molecule is of great interest both structurally and biologically. Once reported, the molecule attracted the attention of many organic synthetic chemists. Baran’s group elegantly achieved the first total synthesis of piperarborenine B in 2011 [78]. In 2016, Hu employed a transition metal-catalyzed [2 + 2] cycloaddition to complete an asymmetric total synthesis of the molecule [115] (Scheme 24).

Hu and co-workers developed a Cu(ClO_4_)_2_-catalyzed intermolecular [2 + 2] cycloaddition reaction paired with **116** as a chiral ligand, starting with compounds **114** and **115** to prepare compound **117** asymmetrically. The use of chiral ligands not only stabilizes organometallic complexes but also introduces a chiral environment during the cyclization process, expanding the application of this strategy to the construction of cyclobutanes. The TBS protection group is removed from adduct **117**, and then it undergoes tandem oxidation and reaction with compound **119** to introduce a directing group, resulting in the formation of compound **120**. Compound **120** subsequently undergoes a C-H arylation reaction under Pd(OAc)_2_ to introduce trimethoxyphenyl, which completes the total synthesis of ( +)-piperarborenine B after several steps of functional group transformations.

**Scheme 25:**
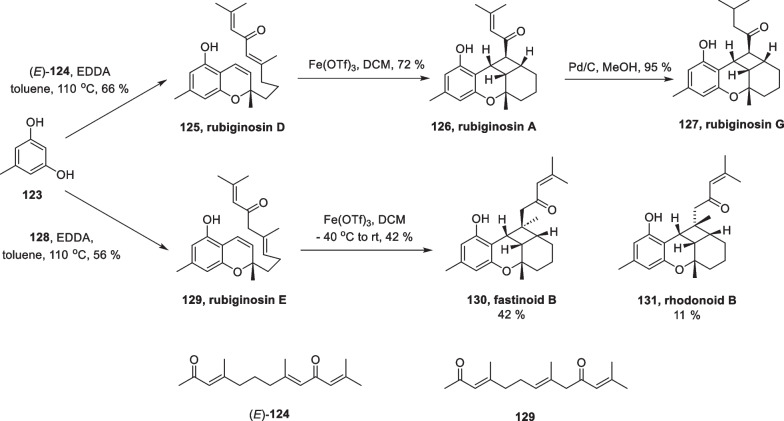
Total Synthesis of six meroterpenoids by Fe-catalyzed [2 + 2] cycloaddition

Besides copper catalyzed [2 + 2] cycloaddition, Fe-catalyzed reactions also play an important role in total synthesis. In 2021, George completed six meroterpenoids rubiginosins A, D, E, and G, fastinoid B, and rhodonoid B. using this method [117] (Scheme 25).

## Non-classical [2 + 2] cycloaddition reactions

During the construction of cyclobutane fragments using [2 + 2] cycloaddition reactions, two olefinic substrate fragments typically interact with each other. This reaction is typically a [2π + 2π] process, where the formation of the two carbon–carbon bonds of the cyclobutane fragment is coupled to each other by interactions between the π electrons. The electrons are then paired to form the σ bonds. In recent years, a novel non-classical [2 + 2] cycloaddition reaction has been widely reported in addition to this bonding mode. The cycloaddition process involves a reaction mechanism where weak chemical bonds within the substrate are broken, releasing the energy of tension. This results in the formation of two alkyl radicals that later undergo a cyclization reaction with another substrate, ultimately leading to the formation of a cyclobutane structure.

**Scheme 26:**
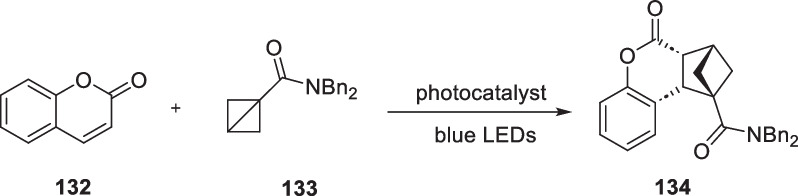
Non-classic intermolecular [2 + 2] cycloaddition between coumarin analogs and high-tensile bicyclo[1.1.0]butane derivatives

In 2022, Frank Glorius reported that they successfully carried out intermolecular [2π + 2σ] cycloaddition reactions between coumarin analogs and high-tensile bicyclo[1.1.0]butanes under light conditions [118]. This reaction leds to the production of a series of bicyclo[2.1.1]hexane compounds with notable medicinal properties (Scheme 26).

The authors observed that bicyclo[1.1.0]butane derivatives possessed multiple high-strength ring systems. Additionally, the sp3 carbon atoms have lost their tetrahedral structure, resulting in a significant degree of carbon–carbon bond bending. As a result, these bonds could be easily broken by external effects. Coumarins have a significant electron delocalization system between the benzene ring and the unsaturated lactone ring. When exposed to ultraviolet light, they can emit fluorescence. The presence of hydroxyl and other chromophores on the benzene ring further enhances the fluorescence. When the compounds are exposed to light in the presence of a photocatalyst, the resulting triplet species have an extended excited state lifetime, facilitating interaction with high-strength rings and tandem cyclization. Through a screening of photocatalysts, solvents and reaction conditions, the compound **134** was obtained by intermolecular [2π + 2σ] cycloaddition reaction. They also synthesized a range of derivatives with different substituent types using this reaction.

**Scheme 27:**
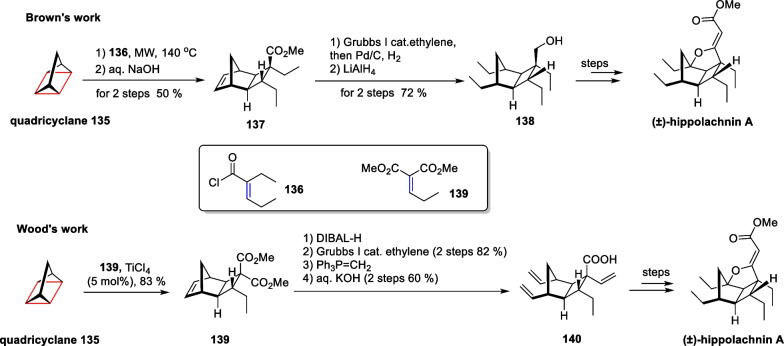
Non-classic intermolecular [2 + 2] cycloaddition in the total synthesis of hippolachnin A

Hippolachnin A has gathered global attention for its unique properties and characteristics [70]. In comparison to Carreira’s previous synthesis approach for hippolachnin A [71], Prof. Wood from Baylor University and Associate Prof. Brown from Indiana University, respectively, developed a novel method using a high-strength ring to successfully complete the total synthesis of this compound [119] (Scheme 27).

Both Wood and Brown utilized tetracyclic [2.2.1.0^2,6^.0^3,5^]heptane as the initial material in the synthesis. They constructed a cyclobutane fragment in the core skeleton of hippolachnin A by performing an intermolecular [2π + 2σ] cycloaddition with polysubstituted olefinic substrates. This process involved breaking weak bonds in the tensile ring under visible light-catalyzed conditions, enabling the creation of four chiral centers in a single step. Subsequent steps included alkene complex metathesis, reductive hydrogenation, and a bridgehead sp3 C-H oxidation, ultimately leading to the successfully total synthesis.

## Conclusions

A variety of cyclobutane-containing natural products featuring peculiar skeletons and diverse activities have been successfully synthesized in recent years. The [2 + 2] cycloaddition is employed as a dominant approach to assemble the cyclobutane scaffolds. Advancements in the field of visible light catalysis, organic catalysis, and transition metal catalysis have significantly enhanced the modes of [2 + 2] cycloaddition reactions. These progressions have led to improved reaction efficiency, increased functional group compatibility, and expanded application scopes for this reaction. The integration of computational chemistry and AI techniques is one of ongoing interest focus for investigating reaction mechanisms. These techniques can be employed to study the reaction mechanism of a specific double bond, estimate the necessary energy for excitation, rationalize photocatalyst screening, and augment stereoselectivity and regioselectivity. With advancements in the [2 + 2] cycloaddition methodology and a deeper comprehension of the cyclobutane chemistry, we anticipate that more efficient synthesis will be developed to assess the structurally complex cyclobutane-containing natural products, thereby facilitating expanded exploration of their chemical space and biological activity.

## Data Availability

All the data and materials provided in the manuscript are obtained from included references and available upon request.
